# Death by neurologic criteria and organ donation in German pediatric intensive care units—data from the PIA network

**DOI:** 10.3389/fped.2026.1890906

**Published:** 2026-10-23

**Authors:** Nadine Mand, Carolin Donath, Franziska König, Sebastian Brenner, Nora Bruns, Christian Dohna-Schwake, Florian Hoffmann, Martin Olivieri, Michael Urschitz, Diana Perera Cutiño, Richard Biedermann

**Affiliations:** 1 Department of Pediatrics, Neonatology and Pediatric Intensive Care Medicine, Marburg University, Marburg, Germany; 2 Department of Pediatrics, Pediatric Intensive Care Medicine, University Clinic Carl Gustav Carus, TU Dresden, Dresden, Germany; 3 Department of Pediatrics I, Neonatology, Pediatric Intensive Care Medicine, and Pediatric Neurology, University Hospital Essen, University of Duisburg-Essen, Essen, Germany; 4 Department of Pediatrics, Klinikum Dritter Orden Munich, Munich, Germany; 5 Pediatric Intensive Care Unit, Dr. von Hauner Children’s Hospital, LMU Munich, Munich, Germany; 6 Division of Pediatric Epidemiology, Institute of Medical Biostatistics, Epidemiology, and Informatics, University Medical Centre of the Johannes Gutenberg-University, Mainz, Germany; 7 Department of Child and Adolescent Medicine, Neonatal and Pediatric Intensive Care Section, University Hospital Jena, Jena, Germany

**Keywords:** brain death, children, epidemiology, organ donation, pediatric intensive care unit

## Abstract

**Introduction:**

Each year, between 250 and 300 children are listed for organ transplantation in Germany, while approximately 30 children donate organs. Despite identified barriers to pediatric organ donation, comprehensive data on characteristics and clinical management of pediatric deaths in German pediatric intensive care units (PICUs), especially among organ donors, remains limited. This study aims to analyze the epidemiology of in-PICU deaths and the characteristics of pediatric organ donors within a multicenter network of German PICUs.

**Methods:**

Retrospective analysis of data anonymously collected in 33 PICUs participating in the Pediatric Intensive Care Admission Network (PIA) between May 1, 2023 and July 31, 2025. Patients included were children older than 27 days and younger than 18 years who died during their PICU stay.

**Results:**

Of 11,095 children recorded, 219 died (overall case-fatality rate:19.7/1,000). Median age at death was 6 years (IQR, 1– 13). Death occurred after withdrawal (30.1%) or limitation of life-sustaining treatment (20.5%), failed maximal therapy (26.5%) and failed cardiopulmonary resuscitation (12.8%). 21/219 (9.6%) children met neurologic criteria for death. Of these, 11 children became organ donors (52.4%). Median age of organ donors was 4 years (IQR 1–13).

**Discussion:**

This first multicenter analysis from a nationwide German PICU network shows comparable mortality rates but substantially lower rates of death by neurologic criteria (DNC) in German PICUs than in international multicenter studies. This gap may indicate missed opportunities for organ donation. More structured donor identification, enhanced training for DNC diagnosis, and development of evidence-based guidelines for pediatric donor management may enhance quality of care and increase pediatric organ donation in Germany.

## 1 Introduction

Advances in prenatal care, neonatal intensive care, and pediatric intensive care medicine have significantly increased survival rates among newborns with congenital malformations and children with chronic or severe acute illnesses (1–5). Subsequently, some of these conditions result in end-stage organ failure, necessitating organ transplantation.

Over the past decade, 250 to 300 children have been listed for organ transplantation in Germany annually, while approximately 30 pediatric organ donations are realized each year (6). Although adult organ transplantation in children is feasible, size mismatches frequently limit this option to older children and adolescents or require organ splitting, which is not feasible for all organs. Furthermore, an older donor age may be associated with a decline in organ quality, particularly due to cardiovascular comorbidities (7, 8).

As donation rates fail to meet transplantation needs, waiting times for organ transplantation increase, resulting in substantial waiting list mortality. With an estimated mortality rate of 20% among patients who do not receive a transplant within one year of being listed, pediatric heart transplant candidates face especially high risks, with infants being at even greater risk (9, 10).

Various barriers contribute to low organ donation rates in children. Notably, donation after cardiac death (DCD), accounting for 32% of total pediatric organ donations in the UK (11), is not permitted in Germany, where death by neurological criteria (donation after brain death, DBD) must be diagnosed, before organ donation can be considered (12). Brain death (i.e., irreversible loss of brain function) must be determined in accordance with the guidelines of the German Medical Association issued under the Transplantation Act (12). Determination requires two appropriately qualified physicians and comprises demonstration of coma, brainstem areflexia and apnea and irreversibility of the condition. Depending on the patient's age and the type and localization of the brain injury, this is done either by a defined observation interval with repeated clinical examinations or by confirmatory ancillary testing (e.g., brain scintigraphy, EEG showing absent potentials, or Doppler ultrasonography of all major brain vessels). Requirements vary according to age, with confirmatory ancillary testing being mandatory for children under the age of three (12).

In 2024, 2,188 infants, children and adolescents under the age of 18 died in Germany (13). Nearly 200 of these deaths resulted from traumatic brain injury, drowning, or hypoxic events such as suffocation or foreign body aspiration (14). These figures suggest potential for increased organ donation through systematic donor identification and determination of irreversible loss of brain function. However, critical knowledge gaps impede efforts to improve pediatric organ donation in Germany, particularly regarding factors distinguishing successful organ donors from other PICU deaths. A systematic analysis of differences in patient characteristics, clinical course, and intensive care interventions between these groups could identify modifiable factors and inform evidence-based strategies to increase organ donation rates. Without such data, developing targeted interventions remains challenging.

In a unique attempt to establish a nationwide database on pediatric critical care in Germany, the Pediatric Intensive Care Unit Admissions (PIA) network was initiated in May 2023 (15). In this study, we aimed to analyze 1) the epidemiology of in-PICU death within the PIA network, 2) the characteristics of patients with organ donation after brain death, and 3) intensive care treatment in pediatric organ donors.

## 2 Methods

### 2.1 Study design and population

A retrospective analysis of data anonymously collected in 33 PICUs participating in the Pediatric Intensive Care Unit Admission (PIA) Network was conducted. Following the initiation of the network with three PICUs in May 2023, the network has grown gradually, with participating PICUs starting data entry at different time points. To date, approximately 80% of participating hospitals are university hospitals or maximum care hospitals, which are located throughout Germany.

A detailed methodology report on the PIA network has been published previously (15). Data are collected retrospectively and anonymously upon PICU discharge via electronic case report forms (eCRF) for eligible patients. A basic survey records all consecutive PICU admissions of patients older than 27 days and younger than 18 years of age and forms the denominator for admission-based rates. A detailed survey is conducted on patients with a PICU length of stay of at least 48 h or in-hospital death within the first two days of admission.

For this analysis, data from May 1, 2023 until July 31, 2025, from all children who died during their PICU stay, irrespective of length of stay, were included. Overall case-fatality rates were calculated using all recorded admissions as the denominator, so that short-stay survivors are also captured. Neonates (27 days of age or younger) are not included in the PIA network by design (15), as this population is predominantly managed in neonatal intensive care units.

### 2.2 Variables

The detailed survey consists of six main sections: 1) Demographics (sex, age) and admission type (planned/unplanned, medical/surgical); 2) admission information, including pre-admission Pediatric Cerebral Performance Category Score (PCPC-Score); 3) organ function within the first hour of admission 4) diagnosis and procedures; 5) interventions during PICU stay; and 6) outcome at PICU discharge. A validated illness-severity score (such as PIM2/PIM3) is not prospectively captured in the PIA eCRF and could not be calculated retrospectively; it was therefore not available for this analysis.

Modes of death in the PICU are mutually exclusive and classified as follows: withdrawal or limitation of life-sustaining treatment (LST), failed maximal therapy, failed cardiopulmonary resuscitation (F-CPR), and death by neurologic criteria (DNC). Withdrawal of LST refers to discontinuation of ongoing life-sustaining measures following a decision to withdraw treatment. Limitation of LST refers to a decision not to initiate or not to escalate life-sustaining measures. F-CPR refers to death despite attempted CPR. Failed maximal therapy refers to death despite ongoing maximal intensive care treatment. DNC refers to death following the determination of irreversible loss of brain function in accordance with the applicable German guidelines. These categories were assigned by the participating PICUs and were not retrospectively adjudicated by the study investigators. Patients for whom the circumstances of death could not be determined were classified as unknown.

### 2.3 Statistical analysis

A descriptive analysis was performed with categorical variables presented as frequencies and percentages, continuous variables as mean and SD, or as median and interquartile range (IQR) whenever normal distribution was not present. The overall case-fatality rate is reported as a proportion with a 95% confidence interval and, additionally, per 1,000 admissions. SAS (version 9.4) was used for statistical analysis.

## 3 Results

### 3.1 Epidemiology of death in the PIA network

Over the 27-month study period, 11,095 children were admitted to participating PICUs. Of these, 219 died, corresponding to an overall case-fatality rate of 19.7 (95% CI, 17.3–22.5) deaths per 1,000 admissions (219/11,095). Two children (0.9%) were declared dead shortly after their arrival at the PICU, while 217 (99.1%) died during their PICU stay. A detailed survey was completed for 4,009 children, comprising all 219 children who died plus 3,790 of the 10,876 surviving children who had a PICU length of stay of at least 48 h (see Figure 1). The median age at death was 6 years (IQR, 1– 13). 44 (20.1%) patients were younger than 1 year, 119 (54.3%) patients were between 1 and 12 years, and 56 (25.6%) patients were older than 12 years.

**Figure 1:**
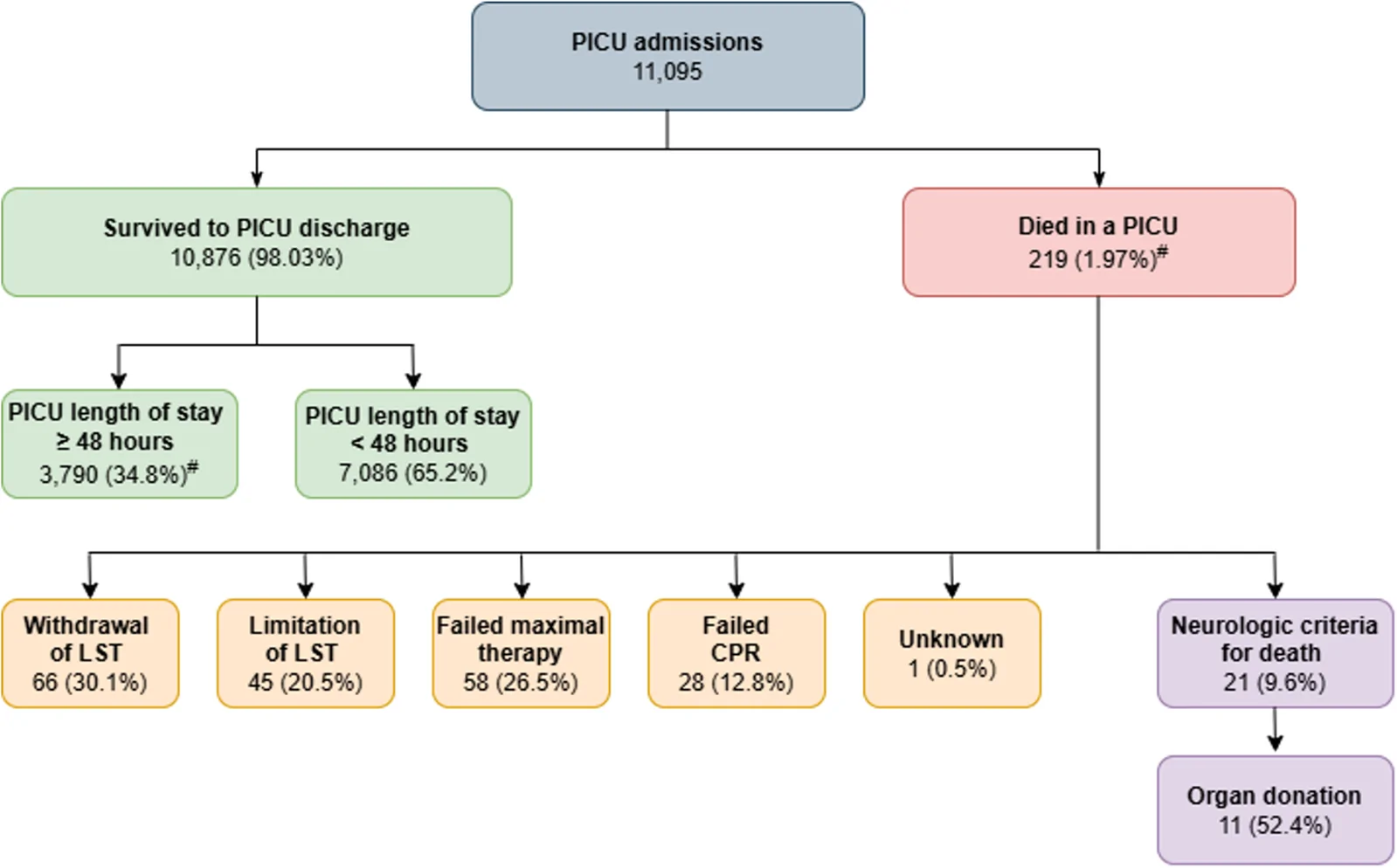
PICU admissions, deaths and mode of death within the PIA network. ^#^detailed survey.

Assessment of pre-admission neurological status using the PCPC revealed that 115 of the 219 children who died (52.5%) had no prior neurological impairment, whereas 22 (10.1%) children had mild, 24 (11.0%) moderate, and 41 (18.7%) severe neurological impairment before being admitted to the PICU.

Details on the initial leading symptoms and underlying causes for the PICU stay are shown in Table 1.

**Table 1:** Characteristics of discharged and deceased children with a detailed survey.

Variable	Discharged or transferred from PICU alive^#^ (*N* = 3,790)	Deceased (*N* = 219)
Age [median (IQR)*]	4 (1–11)	6 (1–13)
LOS^§^ [median (IQR)]	6 (4–11)	4 (2–10)
Neurological impairment pre-admission		
None	2,198 (58.0%)	115 (52.5%)
Mild	471 (12.4%)	22 (10.1%)
Moderate	376 (9.9%)	24 (11.0%)
Severe	487 (12.9%)	41 (18.7%)
Unknown	258 (6.8%)	17 (7.7%)
Leading symptom upon arrival [n (%)]		
Respiratory	1,542 (40.7%)	71 (32.4%)
Cardiovascular	324 (8.5%)	52 (23.7%)
Neurologic	831 (21.9%)	59 (26.9%)
Nephrologic	134 (3.5%)	1 (0.5%)
Gastrointestinal	480 (12.7%)	20 (9.1%)
Dermatologic	25 (0.7%)	0 (0%)
Musculoskeletal	152 (4%)	1 (0.5%)
Hematopoietic/Lymphatic	118 (3.1%)	1 (0.5%)
Other	173 (4.6%)	10 (4.6%)
Unknown	11 (0.3%)	4 (1.8%)
Cause of leading symptom [n (%)]		
Infectious	1,548 (40.8%)	66 (30.1%)
Immunological/Rheumatological	135 (3.6%)	6 (2.7%)
Oncologic/Hematologic	391 (10.3%)	17 (7.8%)
Transplant (solid organs)	43 (1.1%)	4 (1.8%)
Transplant (bone marrow)	40 (1.1%)	10 (4.6%)
Metabolic/Endocrine	138 (3.6%)	8 (3.7%)
Trauma/Surgical	668 (17.6%)	27 (12.3%)
Burns/Scalds/Chemical burns	34 (0.9%)	0 (0%)
Respiratoric/Cardiocirculatoric and	707 (18.7%)	50 (22.8%)
Neurologic		
Unknown	86 (2.3%)	31 (14.2%)

^#^Detailed survey in all children with a length of stay of at least 48 h, 3,790/11,095;*IQR *=* *interquartile range; ^§^LOS* *=* *Length of stay.*

Among the 219 recorded deaths, 66 (30.1%) occurred after withdrawal of life-sustaining treatment (LST), and 45 (20.5%) after limitation of LST. 58 (26.5%) children died after failed maximal therapy, and 28 (12.8%) died after failed cardiopulmonary resuscitation (F-CPR). In one (0.5%) patient, the circumstances of death were unknown. 21 (9.6%) met neurologic criteria for death (see Figure 1 and Table 2).

**Table 2:** Pediatric deaths in PICUs by mode of death.

Variable	Withdrawal of LST^a^ (*n* = 66)	Limitation of LST^a^ (*n* = 45)	Failed CPR^b^ (*n* = 28)	Failed maximal therapy (*n* = 58)	DNC^c^ (*n* = 21)
Age [median (IQR)*]	4.5 (1–12.75)	7 (1–13)	3 (1–12)	6 (2–12)	4 (1–10)
LOS^§^ [median (IQR)]	5 (2–12)	5 (3–17)	1 (1–1)	3 (2–18)	3 (2–6)
Neurological impairment pre-admission					
None	35 (53.0%)	14 (31.1%)	18 (64.3%)	34 (58.6%)	14 (66.7%)
Mild	8 (12.1%)	4 (8.9%)	2 (7.1%)	5 (8.6%)	3 (14.3%)
Moderate	5 (7.6%)	10 (22.2%)	1 (3.6%)	6 (10.3%)	2 (9.5%)
Severe	13 (19.7%)	11 (24.4%)	4 (14.3%)	11 (19.0%)	1 (4.8%)
Unknown	5 (7.6%)	6 (13.3%)	3 (10.7%)	2 (3.4%)	1 (4.8%)
Leading symptom upon arrival [n (%)]					
Respiratory	16 (24.2%)	23 (51.1%)	9 (32.1%)	20 (34.5%)	3 (14.3%)
Cardiovascular	18 (27.3%)	6 (13.3%)	11 (39.3%)	16 (27.6%)	1 (4.8%)
Neurologic	24 (36.4%)	7 (15.6%)	1 (3.6%)	12 (20.7%)	14 (66.7%)
Nephrologic	0 (0%)	0 (0%)	1 (3.6%)	0 (0%)	0 (0%)
Gastrointestinal	4 (6.1%)	9 (20.0%)	1 (3.6%)	6 (10.3%)	0 (0%)
Dermatologic	0 (0%)	0 (0%)	0 (0%)	0 (0%)	0 (0%)
Musculoskeletal	0 (0%)	0 (0%)	0 (0%)	0 (0%)	1 (4.8%)
Hematopoietic/Lymphatic	0 (0%)	0 (0%)	0 (0%)	0 (0%)	1 (4.8%)
Other	4 (6.1%)	0 (0%)	3 (10.7%)	2 (3.4%)	1 (4.8%)
Unknown	0 (0%)	0 (0%)	2 (7.1%)	2 (3.4%)	0 (0%)
Cause of leading symptom [n (%)]					
Infectious	17 (25.8%)	17 (37.8%)	8 (28.6%)	18 (31.0%)	5 (23.8%)
Immunological/Rheumatological	2 (3.0%)	2 (4.4%)	0 (0%)	2 (3.4%)	0 (0%)
Oncologic/Hematologic	5 (7.6%)	2 (4.4%)	1 (3.6%)	7 (12.1%)	2 (9.5%)
Transplant (solid organs)	0 (0%)	0 (0%)	0 (0%)	4 (6.9%)	0 (0%)
Transplant (bone marrow)	1 (1.5%)	4 (8.9%)	0 (0%)	5 (8.6%)	0 (0%)
Metabolic/Endocrine	4 (6.1%)	3 (6.7%)	0 (0%)	1 (1.7%)	0 (0%)
Trauma/Surgical	9 (13.6%)	2 (4.4%)	3 (10.7%)	3 (5.2%)	10 (47.6%)
Burns/Scalds/Chemical burns	0 (0%)	0 (0%)	0 (0%)	0 (0%)	0 (0%)
Respiratoric / Circulatoric / Neurologic	22 (33.3%)	9 (20.0%)	6 (21.4%)	10 (17.2%)	3 (14.3%)1
Unknown	6 (9.1%)	6 (13.3%)	10 (35.7%)	8 (13.8%)	(4.8%)

a LST: Life-sustaining therapy, ^b^CPR: Cardiopulmonary resuscitation, ^c^DNC: Death by neurologic criteria.

### 3.2 Patients with neurologic criteria for death

One patient (4.8%) met neurologic criteria for death upon arrival, 20 (95.2%) during their PICU stay. Of these, 11 patients became organ donors (52.4%; see Table 3).

**Table 3:** Characteristics of children, who subsequently were declared dead by neurologic criteria.

Variable	Death by neurologic criteria WITHOUT organ donation (*N* = 10)	Death by neurologic criteria WITH organ donation (*N* = 11)
Age (median, IQR)	3.5 (1.25–8)	4 (1–13)
LOS	4 (2–5.5)	3 (3–6.5)
Neurological impairment pre-admission		
None	6 (60%)	8 (72.7%)
Mild	2 (20%)	1 (9.1%)
Moderate	1 (10%)	1 (9.1%)
Severe	1 (10%)	0 (0%)
Unknown	0 (0%)	1 (9.1%)
Cause of leading symptom [n (%)]		
Infectious	1 (10%)	4 (36.4%)
Immunologic/Rheumatologic	0 (0%)	0 (0%)
Oncologic/Haematologic	2 (20%)	0 (0%)
Transplant (solid organs)	0 (0%)	0 (0%)
Transplant (bone marrow)	0 (0%)	0 (0%)
Metabolic/Endocrine	0 (0%)	0 (0%)
Trauma/Surgical	5 (50%)	5 (45.4%)
Burns/Scalds/Chemical burns	0 (0%)	0 (0%)
Respiratoric / Cardiocirculatoric / Neurologic	1 (10%)	2 (18.2%)
Unknown	1 (10%)	0 (0%)

The median age of organ donors was 4 years (IQR 1–13). All organ donors had unplanned admissions, primarily for traumatic or surgical causes. Median length of stay on the PICU was three days (IQR 3–6,5); (see Table 3).

### 3.3 Intensive care treatment in organ donors

All patients who became organ donors received mechanical ventilation and cardiovascular support, predominantly for more than 24 h and up to 7 days. The most frequently used vasoactive medications were norepinephrine (100%), milrinone (54.6%), dobutamine (45.5%), and epinephrine (36.4%). Hydrocortisone was administered to 2/11 (18.2%) patients. Extracorporeal treatment was used in two cases (18.2%): one patient received plasmapheresis/immunoadsorption, and another veno-arterial extracorporeal membrane oxygenation (VA-ECMO). No patient received hemodialysis, hemofiltration, or peritoneal dialysis. Sedatives were used in 9 (81.8%), blood products in 10 (90.9%) patients (see Table 4).

**Table 4:** Intensive care treatment in children, who subsequently were declared dead by neurologic criteria.

Variable	Death by neurologic criteria WITHOUT organ donation (*N* = 10)	Death by neurologic criteria WITH organ donation (*N* = 11)
Mechanical ventilation [n (%)]		
≤24hrs	1 (10%)	1 (9.1%)
>24hrs and ≤7d	7 (70%)	7 (63.61%)
>7d and ≤28d	2 (20%)	3 (27.3%)
Cardiocirculatory support [n (%)]*		
Norepinephrine	8 (80%)	11 (100%)
Epinephrine	3 (30%)	4 (36.4%)
Dobutamine	3 (30%)	5 (45.5%)
Dopamine	2 (20%)	1 (9.1%)
Vasopressin	0 (0%)	1 (9.1%)
Milrinone	1 (10%)	6 (54.6%)
Levosimendan	0 (0%)	1 (9.1%)
Akrinor	1 (10%)	0 (0%)
Hydrocortisone	4 (40%)	2 (18.2%)
Others	8 (80%)	7 (63.6%)
Duration of cardiocirculatory support		
≤24hrs	0 (0%)	1 (9.1%)
>24hrs and ≤7d	7 (70%)	8 (72.7%)
>7d and ≤28d	3 (30%)	2 (18.2%)
>28d	0 (0%)	0 (0%)
Unknown	1 (10%)	0 (0%)
Extracorporeal treatment*		
Plasmapheresis/Immunoadsorption	0 (0%)	1 (9.1%)
VA-ECMO	0 (0%)	1 (9.1%)
Others	0 (0%)	2 (18.2%)
Sedatives*		
Opioids	6 (60%)	9 (81.8%)
Benzodiazepines	4 (40%)	4 (36.4%)
Propofol	1 (10%)	7 (63.6%)
Ketamin/Esketamin	0 (0%)	1 (9.1%)
Volatile agents	0 (0%)	1 (9.1%)
Alpha-agonists	1 (10%)	1 (9.1%)
Others	6 (60%)	6 (54.6%)
Duration of Sedation		
≤24hrs	3 (30%)	3 (27.3%)
>24hrs and ≤7d	4 (40%)	3 (27.3%)
>7d and ≤28d	0 (0%)	2 (18.2%)
Unknown	3 (30%)	3 (27.3%)
Blood products*		
Erythrocyte concentrates	3 (30%)	6 (54.6%)
Thrombocyte concentrate	1 (10%)	2 (18.2%)
Fresh Frozen Plasma	4 (40%)	4 (36.4%)
Coagulation factors	2 (20%)	5 (45.5%)
Tranexamid Acid	1 (10%)	0 (0%)
Albumin	2 (20%)	1 (9.1%)
Fibrinogen	1 (10%)	3 (27.3%)
IVIG	2 (20%)	1 (9.1%)
Others	4 (40%)	6 (54.6%)

*Multiple answers possible.

## 4 Discussion

This study provides the first comprehensive analysis of death in pediatric patients in German PICUs and of patients who became pediatric organ donors through evaluation in the German Pediatric Intensive Care Unit Admission (PIA) Network. The retrospective analysis of 11,095 admissions across 33 PICUs identified 219 deaths, yielding a case fatality rate of 19.7/1,000 (95% CI 17.2–22.5) consistent with other high-income countries (18–27/1,000) (17–20).

Our cohort's median age at death (6 years, IQR 1–13) was higher than in previous reports (16, 20). Only 20% of deaths occurred in infancy, with a further 25% occurring in teenagers. This age distribution may reflect differences in PICU admission or referral patterns in Germany. Approximately 50% of deaths were due to neurological, respiratory, cardiovascular, and infectious conditions. This proportion is lower than that observed in other international cohorts (20), possibly due to an older age structure, lower pre-ICU arrest rates and a high proportion of deaths (14.2%) due to an unknown underlying condition.

It is notable that more than half of the deceased children (52.5%) had no neurological impairments prior to being admitted to the PICU. However, data on other non-neurological chronic conditions that often contributed to pediatric mortality in previous studies (17, 20, 21) is lacking. Among those with abnormal pre-admission PCPC scores, almost half had severe neurological impairment. This may have had a certain degree of influence on end-of-life decisions for some of these children. Decisions to limit life-sustaining treatment are particularly prevalent among children with severe neurological impairments and can have a significant impact on mortality rates (18, 20).

Half of the deaths (50.6%) occurred following withdrawal or limitation of LST, aligning with reported European rates (18, 22) but slightly lower than those reported in non-European multicenter studies (63%–70%) (20, 21, 23). Unfortunately, we were unable to provide a full characterization of end-of-life practices, as information on DNR orders, the severity of illness prior to LST decisions, and the rationale for LST was not available. Additionally, our data is unable to determine the number of children who may have undergone DNC evaluation following the decision to limit LST. In this study, only 21 of 219 deaths (9.6%) were attributed to DNC, which is lower than the 15%–23% reported in international multicenter studies (18, 23–26). There are several possible factors contributing to this low rate: structural barriers (e.g., a lack of standardized protocols for identifying potential DNC), organizational limitations (e.g., insufficient resources for the time- and labor-intensive protocolized DNC evaluation) and limited clinical expertise in diagnosing pediatric DNC have been stated as potential barriers in the literature (27, 28). The stringent, age-specific requirements for determining DNC in Germany, including mandatory ancillary testing in the youngest children (12), may further contribute to this comparatively low rate. Further studies have to address those potential barriers for DNC evaluation in potential pediatric organ donors in Germany.

Among the 21 patients who met the neurologic criteria of death, 11 (52.4%) donated organs. As it is unclear whether all 21 families were approached for organ donation, we are unable to specify a family consent rate. However, other national multicenter studies in North America have documented consent rates of approximately 50% in pediatric organ donation (25, 26), while in German adult patients consent rates of less than 25% have been reported (29). Potential barriers for non-donation previously identified include challenges in identifying and reporting potential donors to the relevant authorities (30–32), religious concerns, perceived medical contraindications, a reluctance to approach families of young children (33, 34), or lower consent rates for infants and toddlers (33, 35) - though age distribution was similar in our donor and non-donor group. Notably, five patients admitted for trauma did not donate organs which suggests barriers beyond medical suitability. Research indicates that healthcare professionals' attitudes and experiences can significantly impact on end-of-life care (36, 37) and organ donation consent rates for patients with neurological criteria for death (38). In our cohort, the specific reasons for donating or not donating organs remain unclear and need to be addressed in future research.

The complexity of maintaining physiological stability in young potential donors—particularly those under two years—poses yet another challenge to successful organ donation (24), as reflected by the intensive use of vasoactive agents and blood products in DNC patients who became donors in our cohort. Intensive care management of potential donors demands substantial resources, which may not be possible in some cases, potentially also impacting organ donation rates. Moreover, evidence based protocols on optimal intensive care management of potential pediatric organ donors remain limited. Most recommendations are based on established pediatric intensive care treatment principles or are derived from adult studies as ‘good clinical practice’ (39). Finally, regional variability in PICU structures and processes likely contribute to differences in donation practices, further complicating standardization (40).

**Strengths** of this study include the large, geographically diverse national cohort and the systematic capture of PICU deaths within a network in which most pediatric deaths and all DNC diagnoses occur. This provides a robust overview of circumstances surrounding pediatric death and organ donation in Germany. **Limitations** include potential reporting bias of participating PICUs in the PIA network, heterogeneous practices across PICUs, staggered network enrollment affecting temporal coverage, exclusion of the neonatal population, the absence of a validated illness-severity score (such as PIM2/PIM3) and the lack of granular data on LST decision-making and on the DNC diagnostic process.

**Conclusion** This first multicenter analysis from a nationwide German PICU network reveals that German PICUs have comparable mortality rates but substantially lower DNC diagnosis rates than international centers. This gap might suggest missed opportunities for organ donation and has to be addressed in future research. The findings highlight the need for a systematic review of all PICU deaths for donation eligibility, protocols for identifying and evaluating of potential DNC cases, enhanced training for DNC diagnosis, and development of evidence-based guidelines for pediatric donor management. There is an urgent need for national quality-improvement initiatives targeting end-of-life care and organ donation processes. These interventions could improve pediatric organ donation rates, potentially saving the lives of children awaiting organ transplantation.

## Data Availability

The datasets presented in this article are not readily available because avaiability of data sets is not planned. Requests to access the datasets should be directed to mandn@staff.uni-marburg.de.

## A group members of the PIA study group

Ali Moussa, Andreas Wemhöner, Annika Ander, Annika Wessel, Axel Heep, Beatrice Grebe, Christian Spang, Christoph Härtel, Christopher Meudt, Daniel Acero Moreno, Ellen Heimberg, Fabian Bärtling, Frederike Terpe, Gert Warncke, Guido Stichtenoth, Holger Freymann, Jan Baier, Jeremy Rott, Jochen Essers, Johannes Eisen, Judith Jochem, Kai Fiedler, Lisette Hayer, Marc Hoppenz, Mark Dzietko, Markos Tomidis, Maximilian Groffmann, Michael Merker, Nobert Teig, Ortraud Behringer, Patrizia Nitsch-Felsecker, Pia Paul, Sabine Schäfer, Sarah Irlbeck, Stefanie Hort, Steven Hébert, Tanja Greimel, Thomas Bösing, Uwe Höppner, Vera Wentzel.

## References

[1] Arantes RR Aguiar MB Cunha KCCMS Amaral AA Melo JRO Vieira BCC. Risk factors for perinatal and neonatal mortality in cases with congenital anomalies of the kidney and urinary tract: a nested cohort study. Pediatr Nephrol. (2025) 40:3697–705. 10.1007/s00467-025-06922-y 40783475

[2] Keil C Bedei I Sommer L Koemhoff M Axt-Fliedner R Köhler S. Fetal therapy of LUTO (lower urinary tract obstruction)—a follow-up observational study. J Matern Fetal Neonatal Med. (2022) 35(25):8536–43. 10.1080/14767058.2021.1988562 34652254

[3] Keil C. Global, regional, and national burden of congenital heart disease, 1990–2017: a systematic analysis for the global burden of disease study 2017. Lancet Child Adolesc Health. (2020) 4(3):185–200. 10.1016/S2352-4642(19)30402-X 31978374 PMC7645774

[4] O'Connor M Adler JT Venardos NM Ponce-Rivera MS Fraser CD Mery CM. Outcomes of liver transplantation in patients with congenital heart disease and biliary atresia. A multicenter analysis. Pediatr Transplant. (2025) 29(5):e70110. 10.1111/petr.70110 PMC12125496 40448280

[5] Weiss SL Peters MJ Alhazzani W Agus MSD Flori HR Inwald DP. Surviving sepsis campaign international guidelines for the management of septic shock and sepsis-associated organ dysfunction in children. Pediatr Crit Care Med. (2020) 21(2):e52–e106. 10.1097/PCC.0000000000002198 32032273

[6] German Organ Procurement Organization (DSO). (2025). Jahresbericht 2022. Available online at: https://www.dso.de/SiteCollectionDocuments/DSO-Jahresbericht%202022.pdf (Accessed September 07, 2025).

[7] Ettenger R Venick RS Gritsch HA Alejos JC Weng PL Srivastava R. Deceased donor organ allocation in pediatric transplantation: a historical narrative. Pediatr Transplant. (2023) 27(Suppl 1):e14248. 10.1111/petr.14248 36468338

[8] Broyer M Otte JB Kachaner J Goulet O. Organ transplantation in children. Intensive Care Med. (1989) 15(Suppl 1):S76–9. 10.1007/BF00260895 2656818

[9] Butts RJ Toombs L Kirklin JK Schumacher KR Conway J West SC. Waitlist outcomes for pediatric heart transplantation in the current era: an analysis of the pediatric heart transplant society database. Circulation. (2024) 150(5):362–73. 10.1161/CIRCULATIONAHA.123.068189 38939965

[10] Mah D Singh TP Thiagarajan RR Gauvreau K Piercey GE Blume ED. Incidence and risk factors for mortality in infants awaiting heart transplantation in the USA. J Heart Lung Transplant. (2009) 28(12):1292–8. 10.1016/j.healun.2009.06.013 19782580 PMC4269350

[11] Weiss MJ Domínguez-Gil B Lahaie N Nakagawa TA Scales A Hornby L. Development of a multinational registry of pediatric deceased organ donation activity. Pediatr Transplant. (2019) 23(3):e13345. 10.1111/petr.13345 30724003

[12] Bundesaerztekammer. Beschluss der Bundesärztekammer über die Richtlinie gemäß § 16 Abs. 1 S. 1 Nr. 1 TPG für die Regeln zur Feststellung des Todes nach § 3 Abs. 1 S. 1 Nr. 2 TPG und die Verfahrensregeln zur Feststellung des endgültigen, nicht behebbaren Ausfalls der Gesamtfunktion des Großhirns, des Kleinhirns und des Hirnstamms nach § 3 Abs. 2 Nr. 2 TPG, Fünfte Fortschreibung. Dtsch Arztebl International. 2022;119(35-36):-8513-.

[13] Federal Statistical Office of Germany. (2025). Death Statistics in 2024. Available online at: https://www.destatis.de/DE/Themen/Gesellschaft-Umwelt/Bevoelkerung/Sterbefaelle-Lebenserwartung/Publikationen/Downloads-Sterbefaelle/statistischer-bericht-sterbefaelle-5126105247005.html?templateQueryString = Tod (Accessed August 23, 2025).

[14] Federal Statistical Office of Germany. (2024). Causes of Deaths in Germany in the Year 2023. Available online at: https://www.destatis.de/DE/Themen/Gesellschaft-Umwelt/Gesundheit/Todesursachen/Publikationen/Downloads-Todesursachen/statistischer-bericht-todesursachen-2120400237005.html?templateQueryString = todesursachen + nach + alter (Accessed August 23, 2025).

[15] Bruns N Dohna-Schwake C Olivieri M Urschitz MS Blomenkamp S Frosch C. Pediatric intensive care unit admissions network-rationale, framework and method of operation of a nationwide collaborative pediatric intensive care research network in Germany. Front Pediatr. (2024) 11:1254935. 10.3389/fped.2023.1254935 38269291 PMC10806156

[16] Hamrin T H Eksborg S. Risks for death after admission to pediatric intensive care (PICU)-A comparison with the general population. PLoS One. (2022) 17(10):e0265792. 10.1101/2022.03.09.22272138 36206205 PMC9543762

[17] Killien EY Keller MR Watson RS Hartman ME. Epidemiology of intensive care admissions for children in the US from 2001 to 2019. JAMA Pediatr. (2023) 177(5):506–15. 10.1001/jamapediatrics.2023.0184 36972043 PMC10043802

[18] Agra-Tuñas C Rodriguez-Ruiz E Rodríguez Merino E. How do children die in PICUs nowadays? A multicenter study from Spain. Pediatr Crit Care Med. (2020) 21(9):e610–e6. 10.1097/PCC.0000000000002359 32343103

[19] Viksten J L Engerström L Steinvall I Samuelsson A Fredrikson M Walther S. Children aged 0–16 admitted to Swedish intensive care units and paediatric intensive care units showed low mortality rates. Acta Paediatr. (2019) 108(8):1460–6. 10.1111/apa.14708 30582755

[20] Moynihan KM Alexander PMA Schlapbach LJ Millar J Jacobe S Ravindranathan H. Epidemiology of childhood death in Australian and New Zealand intensive care units. Intensive Care Med. (2019) 45(9):1262–71. 10.1007/s00134-019-05675-1 31270578

[21] Meert KL Keele L Morrison W Berg RA Dalton H Newth CJL. End-of-life practices among tertiary care PICUs in the United States: a multicenter study. Pediatr Crit Care Med. (2015) 16(7):e231–8. 10.1097/PCC.0000000000000520 26335128 PMC4562059

[22] Devictor DJ Latour JM. Forgoing life support: how the decision is made in European pediatric intensive care units. Intensive Care Med. (2011) 37(11):1881–7. 10.1007/s00134-011-2357-3 21965096

[23] Burns JP Sellers DE Meyer EC Lewis-Newby M Truog RD. Epidemiology of death in the PICU at five U. S. Teaching Hospitals*. Crit Care Med. (2014) 42(9):2101–8. 10.1097/CCM.0000000000000498 24979486 PMC4134743

[24] Bonetto G Taffarel P Gamerman M Jorro Barón F Gaviña C Flores L. Brain death and organ donation in Argentine pediatric intensive care units. A multicenter study. Arch Argent Pediatr. (2018) 116(1):e54–60. 10.5546/aap.2018.eng.e54 29333823

[25] Kirschen MP Francoeur C Murphy M Traynor D Zhang B Mensinger JL. Epidemiology of brain death in pediatric intensive care units in the United States. JAMA Pediatr. (2019) 173(5):469–76. 10.1001/jamapediatrics.2019.0249 30882855 PMC6503509

[26] Joffe AR Shemie SD Farrell C Hutchison J McCarthy-Tamblyn L. Brain death in Canadian PICUs: demographics, timing, and irreversibility. Pediatr Crit Care Med. (2013) 14(1):1–9. 10.1097/PCC.0b013e31825b5485 23132398

[27] Greer DM Shemie SD Lewis A Torrance S Varelas P Goldenberg FD. Determination of brain death/death by neurologic criteria: the world brain death project. Jama. (2020) 324(11):1078–97. 10.1001/jama.2020.11586 32761206

[28] Toulouse J Hully M Brossier D Viallard M-L De Saint Blanquat L Renolleau S. The role of the neuropediatrician in pediatric intensive care unit: diagnosis, therapeutics and major participation in collaborative multidisciplinary deliberations about life-sustaining treatments' Withdrawal. Eur J Paediatr Neurol. (2019) 23(1):171–80. 10.1016/j.ejpn.2018.09.002 30262235

[29] Englbrecht JS Schrader D Kraus H Schäfer M Schedler D Bach F. How large is the potential of brain dead donors and what prevents utilization? A multicenter retrospective analysis at seven university hospitals in north rhine-westphalia. Transpl Int. (2023) 36:11186. 10.3389/ti.2023.11186 37252613 PMC10211426

[30] Ekinci F Yıldızdaş D Horoz ÖÖ İncecik F. Evaluation of pediatric brain death and organ donation: 10-year experience in a pediatric intensive care unit in Turkey. Turk Arch Pediatr. (2021) 56(6):638–45. 10.5152/TurkArchPediatr.2021.21130 35110065 PMC8849511

[31] Tackmann E Kurz P Dettmer S. Attitudes and knowledge about post-mortem organ donation among medical students, trainee nurses and students of health sciences in Germany: a cross-sectional study. Anaesthesist. (2020) 69(11):810–20. 10.1007/s00101-020-00812-8 32696082 PMC8580913

[32] Tackmann E Dettmer S. Measures influencing post-mortem organ donation rates in Germany, The Netherlands, Spain and the UK: a systematic review. Anaesthesist. (2019) 68(6):377–83. 10.1007/s00101-019-0600-4 31101922

[33] Godown J Butler A Lebovitz DJ Chapman G. Predictors of deceased organ donation in the pediatric population. Pediatrics. (2021) 147(6). 10.1542/peds.2020-009506 33963074 PMC8785750

[34] De Freitas RA Dell'Agnolo CM Alves EF Benguella EA Pelloso SM Carvalho MD. Organ and tissue donation for transplantation from fatal trauma victims. Transplant Proc. (2015) 47(4):874–8. 10.1016/j.transproceed.2015.03.026 26036476

[35] Bennett EE Sweney J Aguayo C Myrick C Antommaria AH Bratton SL. Pediatric organ donation potential at a Children's Hospital. Pediatr Crit Care Med. (2015) 16(9):814–20. 10.1097/PCC.0000000000000526 26237656

[36] Zanin A Brierley J Latour JM Gawronski O. End-of-life decisions and practices as viewed by health professionals in pediatric critical care: a European survey study. Front Pediatr. (2023) 10:1067860. 10.3389/fped.2022.1067860 36704131 PMC9872024

[37] Williams MA Lipsett PA Rushton CH Grochowski EC Berkowitz ID Mann SL. The physician’s role in discussing organ donation with families. Crit Care Med. (2003) 31(5):1568–73. 10.1097/01.CCM.0000063090.21056.A6 12771634

[38] Rodrigue JR Cornell DL Howard RJ. Pediatric organ donation: what factors most influence parents' Donation decisions? Pediatr Crit Care Med. (2008) 9(2):180–5. 10.1097/PCC.0b013e3181668605 18477931 PMC2410299

[39] Lücking K Seidemann K Hoffmann F. (2021). Exkurs: Intensivtherapie des pädiatrischen Organspenders. Praxisleitfaden Organspende: Umsetzung der BÄK-Richtlinie „Spendererkennung“ in der Praxis. Berlin: Medizinisch Wissenschaftliche Verlagsgesellschaft.

[40] Godown J McKane M Wujcik K Mettler BA Dodd DA. Expanding the donor pool: regional variation in pediatric organ donation rates. Pediatr Transplant. 2016;20(8):1093–7. 10.1111/petr.12779 27507803

